# Exploring the difference between men and women in physical functioning: How do sociodemographic, lifestyle- and health-related determinants contribute?

**DOI:** 10.1186/s12877-022-03216-y

**Published:** 2022-07-22

**Authors:** Lena Dirkje Sialino, H. Susan J. Picavet, Hanneke A. H. Wijnhoven, Anne Loyen, W. M. Monique Verschuren, Marjolein Visser, Laura S. Schaap, Sandra H. van Oostrom

**Affiliations:** 1 Department of Health Sciences, Faculty of Science, Amsterdam Public Health Research Institute, Vrije Universiteit Amsterdam, Amsterdam, The Netherlands; 2 Centre for Nutrition, Prevention and Health Services, National Institute for Public Health and the Environment, Bilthoven, the Netherlands; 3 Julius Centre for Health Sciences and Primary Care, University Medical Centre, Utrecht, The Netherlands

**Keywords:** Physical aging, Life-course, Socio-demographic factors, Health-related factors, Lifestyle factors

## Abstract

**Background:**

To explore whether differences between men and women in the sensitivity to (strength of the association) and/or in the exposure to determinants (prevalence) contribute to the difference in physical functioning, with women reporting more limitations.

**Methods:**

Data of the Doetinchem Cohort Study was used (n = 5856, initial ages 26–70 years), with follow-up measurements every 5 years (up to 20). Physical functioning (subscale SF-36, range:0–100), sex (men or women) and a number of socio-demographic, lifestyle- and health-related determinants were assessed. Mixed-model multivariable analysis was used to investigate differences between men and women in sensitivity (interaction term with sex) and in exposure (change of the sex difference when adjusting) to determinants of physical functioning.

**Results:**

The physical functioning score among women was 6.55 (95%CI:5.48,7.61) points lower than among men. In general, men and women had similar determinants, but pain was more strongly associated with physical functioning (higher sensitivity), and also more prevalent among women (higher exposure). The higher exposure to low educational level and not having a paid job also contributed to the lower physical functioning score among women. In contrast, current smoking, mental health problems and a low educational level were more strongly associated with a lower physical functioning score among men and lower physical activity and higher BMI were more prevalent among men.

**Conclusions:**

Although important for physical functioning among both men and women, our findings provide no indications for reducing the difference in physical functioning by promoting a healthy lifestyle but stress the importance of differences in pain, work and education.

**Supplementary Information:**

The online version contains supplementary material available at 10.1186/s12877-022-03216-y.

## Background

Physical functioning involves the ability of performing daily physical and routine activities. Self-reported data of physical functioning are commonly used for health and ageing research and one common finding is that women more often report limitations in performing these routine activities compared to men [1–4]. Insights into why men and women differ in physical functioning may provide relevant information for the prevention of physical limitations and the promotion of healthy ageing. One approach to study the difference in physical functioning between men and women is to examine differences in (modifiable) determinants for poorer physical functioning. Men and women may differ in the *sensitivity* to determinants; the strength of the association with physical functioning differs. Another possibility is that men and women may differ in their *exposure* to determinants, i.e. the prevalence of determinants differs.

Potential (modifiable) determinants for physical functioning among older adults are socio-demographic, lifestyle-related or health-related [2, 3, 5]. A difference between men and women in the sensitivity to these determinants are often not reported in the literature, but a difference in the prevalence (i.e. exposure) are more commonly known. Socio-demographic factors such as a low level of education, not having a paid job or a low level of social contacts are mentioned as determinants for poorer physical functioning [2, 3, 5], with women on average having a lower level of education [6], more often being unemployed [6] and having more social contacts [7]. Lifestyle related determinants for poorer physical functioning are smoking, physical inactivity, no alcohol consumption compared to moderate consumption and both over- and underweight [2, 3, 5]. For some of these determinants it is known that men and women in western societies differ in exposure, this holds e.g. for smoking (higher among men [8]), alcohol consumption (higher among men [9]), and overweight/obesity (higher among middle-aged men [10]). In addition, many health conditions can be viewed as determinants for poorer physical functioning including all chronic diseases and pain. For a long time it has been described that women are more likely to experience health conditions associated with high morbidity (including worse physical functioning) and low mortality, whereas men are more likely to experience conditions associated with high mortality [11]. Women have a higher life expectancy and a higher prevalence of chronic health conditions [12], with for instance pain more often found among women [13]. So, differences between men and women in exposure to determinants are thus known or to be expected. These are however usually not taken into account while studying determinants for poor physical functioning.

Taken together, the investigation of the difference between men and women in physical functioning is limited, especially using a systematic approach across a wide range of determinants. Therefore, in order to provide insight into how differences between men and women in sensitivity and/or exposure to (modifiable) determinants contribute to the more reported limitations in physical functioning among women compared to men, data from a Dutch cohort study were examined with 20 years of follow-up, representing a population aging from 26–70 to 46–87 years.

## Methods

### Study sample

Data from the Doetinchem Cohort Study were used, a Dutch prospective population-based study [14]. The first cycle (years: 1987–1991) was carried out among 12,406 men and women aged 20–59 years from Doetinchem. A random sample of these respondents (n = 7769) was re-invited every five years. Measurements included questionnaires and a physical examination. The study was conducted according to the principles of the World Medical Association Declaration of Helsinki and its amendments since 1964, and in accordance with the Medical Research Involving Human Subject Act (WMO). All participants gave written informed consent. Physical functioning was measured from 1995 onwards, so the baseline measurement of the study sample was set in the years 1995–1999, with a maximum of four follow-up measurements (supplementary Fig. 1). Participants were included in the current study if they had a physical functioning score and determinant value on at least one measurement point, resulting in 5856 participants (of the 6391), aged 26–70 years at baseline, with on average 2.8 follow-up measurements in the final multivariable model (total number of observations = 16,773).

### Measures

Physical functioning was measured using the physical functioning scale of the Dutch version of the 36-Item Short Form Health Survey (SF-36) [15, 16], which contains responses on the question ‘The following items are about activities you might do during a typical day. Does your health now limit you in these activities? If so, how much?' for the following 10 items: Vigorous activities such as running, lifting heavy objects or participating in strenuous sports; Moderate activities such as moving a table, vacuuming or bicycling; lifting or carrying groceries; climbing several flights of stairs; climbing one flight of stairs; bending, kneeling, or stooping; walking more than a thousand meter; walking 500 m; Walking 100 m and bathing or dressing yourself. The response options were: “yes, limited a lot”/”yes, limited a little”/”no, not limited at all”. The sum scores were rescaled to a score from 0 to 100, where higher scores indicate better physical functioning [16].

Possible determinants included sociodemographic and lifestyle- and health-related characteristics. Sociodemographic characteristics included sex (sex registered at civil registers, i.e. defined at birth), age (years), work status (yes/no), living alone (yes/no) and level of education (defined as the highest level of education obtained). Educational level was categorized into low (intermediate secondary education or less), moderate (intermediate vocational or higher secondary education), and high (higher vocational education or university). Lifestyle characteristics included current smoking status (yes/no), alcohol consumption (total number of glasses alcohol per day, categorized into ≤ 1 glass/day and > 1 glass/day), physical activity (assessed with a self-administered questionnaire on time spent on physical activities, defined as total hours/week including light, moderate or vigorous leisure time physical activities, sleep duration (average per 24 h, and classified in short (≤ 6 h/night), normal (7–8 h/night) and long (≥ 9 h/night) [17]) and BMI (Body Mass Index), calculated by weight(kg)/height(m)^2 from measured height and body weight (with classification following WHO guidelines [10]). Health-related characteristics included self-reported chronic conditions, pain and mental health. The number of chronic conditions was based on the following diseases: stroke, heart attack, diabetes, cancer, and chronic low back pain. Pain was based on the question “How much bodily pain have you had during the past 4 weeks?” part of the SF-36 questionnaire with response options “none”, “very mild”, “mild”, “moderate”, “severe” and “very severe” [16]. “Very mild” and “mild” were combined as mild pain, and the higher categories in ‘moderate to severe’. Mental health was measured using the Mental Health Inventory (MHI-5) and dichotomized into ‘poor’ (≤ 60) versus ‘good’ (> 60) mental health [18].

### Data analyses

**Fig. 1:**
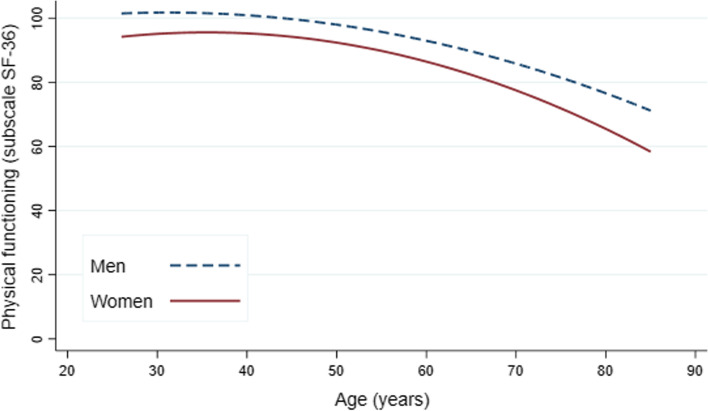
The unadjusted longitudinal course of physical functioning by age, for men (blue dashed) and women (red solid). Modelled using a tobit model, predicted values may exceed the maximum score of 100

Multivariable mixed model analyses for repeated measures was used, including sociodemographic, lifestyle and health-related determinants. A tobit model in the likelihood function was used to correct for the maximum threshold of 100 of the SF-36 physical functioning score that is accompanied with a ceiling effect [19, 20]. The model with the best fit to depict the difference between men and women in physical functioning by age also included age^2^. Multicollinearity among determinants was tested and not found (all variance inflation factors < 10 and correlation < 0.5). In order to evaluate the differences between men and women in sensitivity we examined the interaction term determinant*sex separately for each determinant in a multivariable model including all determinants. A p < 0.10 belonging to this interaction beta was considered as a statistical significant difference in sensitivity, i.e. the association between the determinant and physical functioning [21]. Each multivariable association between the determinant and physical functioning was investigate for men and women separately. In order to evaluate the difference between men and women in exposure we determined the percentage change of the beta sex in physical functioning by adjustment for the determinant of interest [22]. A relevant percentage change was set at > 5%. Because our data presents a wide age range we also explored whether the findings were different for those under and over 55 years of age at baseline. This statistical cut-off was chosen to create similar groups with regard to size and number of repeated measurements and to define the age after which the difference in physical functioning between men and women increases (Fig. 1).

#### Results

### Baseline characteristics

There were 5856 participants with at least one measurements of physical functioning; 2762 men and 3094 women Table 1. At baseline, participants were between 26 and 70 years old. The age-unadjusted mean of the physical functioning score was higher for men (89.5) than women (86.0). Compared to men, women had on average a lower level of education, higher level of physical activity, were less often employed, reported more pain—both ‘mild’ and ‘moderate to severe’- and a lower score on mental health. In addition, alcohol consumption (> 1 glass/day) and overweight were more often found among men.

**Table 1:** Baseline characteristics of the men and women of the study population (*n* = 5856)

**Population** N (%)		**Men**	**Women**
		2762 (47.0)	3094 (53.0)
Age	Mean (SD)	47.9 (10.5)	46.9 (10.6)
Physical functioning^a^	Mean score (SD)	89.5 (15.8)	86.0 (18.0)
**Socio-demographic determinants**			
Level of education^b^	% Low	39.4	55.2
	% Medium	34.3	26.3
	% High	26.4	18.6
Work status	% Unemployed	24.4	52.1
Living arrangements	% Living alone	6.7	9.2
**Lifestyle-related determinants**			
Alcohol consumption	% ≤ 1 glass/day	46.6	78.5
	% > 1 glass/day	53.4	21.5
Current smoking status	% Current Smoker	29.4	29.8
Physical activity (h/week)	Mean (SD)	28.8 (21.7)	39.7 (20.4)
Sleep duration	% Short (≤ 6 h/night)	19.9	13.7
	% Normal (7–8 h/night)	74.2	78.4
	% Long (≥ 9 h/night)	5.9	7.9
BMI (kg/m2)	Mean (SD)	26.0 (3.3)	25.4 (4.3)
	% Underweight (BMI < 18)	0.2	0.6
	% Normal weight (18 ≤ BMI < 25)	39.3	50.3
	% Overweight (25 ≤ BMI < 30)	47.2	33.3
	% Obese (BMI ≥ 30)	13.4	15.8
**Health-related determinants**			
Chronic diseases	% None	59.3	56.5
	% One disease	36.9	40.5
	% ≥ 2 diseases	3.8	3.0
Pain	% None	50.9	39.1
	% Mild	35.8	40.9
	% Moderate to severe	13.3	20.0
Mental health (MHI-5)	% Poor mental health (< 60)	13.1	20.3

*Abbreviations: SD Standard Deviation, BMI Body Mass Index*

^a^ Physical functioning scale of the Dutch version of the 36-item Short Form Health Survey (SF-36)

^b^ Low = elementary, lower vocational or general intermediate education; Medium = intermediate vocational, general secondary or higher vocational education; High = college education and university

### Sex difference in physical functioning by age

Physical functioning decreased with increasing age for both men and women Fig. 1 and Table 2. Adjusted for age, women scored on average 6.55 (95%CI:5.48,7.61) points lower than men (on a scale from 0 to 100) (Table 2). The sex difference is shown at all ages, though the gap seems to widen at older ages, from on average of 6 points at age 45 to 12 points at age 80. Both the linear (Beta = -0.54) and quadratic (Beta = -0.01) change in the sex difference were statistically significant, (p < 0.001 and p < 0.001) (Table 2).

**Table 2:** Difference between men and women in the longitudinal course of physical functioning, statistics belonging to Fig. 1

**Variable**	**Beta**	**95%CI**		***P*-value**
Age	-0.54	(-0.57	-0.50)	< 0.001
+ Age * Age	-0.01	(-0.01	-0.01)	< 0.001
+ Sex (women)	-6.55	(-7.61	-5.48)	< 0.001
+ Age * Sex (women)	-0.09	(-0.15	-0.03)	0.006
+ Age * Age * Sex (women)	-0.00	(-0.01	-0.00)	0.010

*Abbreviations: 95%CI* = *95% Confidence Interval*

*Note:* each row represents an additional added variable ( +) to the model

### Differences between men and women in sensitivity

For both men and women, chronic diseases and pain were the strongest determinants associated with physical functioning Table 3. There was a difference in sensitivity between men and women – looking at the interaction terms of determinant*sex and comparing the associations for men and women separately – to current smoking, pain, mental health problems and educational level (p < 0.10). Mild pain had a larger negative association with physical functioning among women, and current smoking, a low educational level and mental health problems had a larger negative association with physical functioning among men. To illustrate: Mild versus no pain was associated with a lower physical functioning score of 10.9 points (95%CI:11.8,9.87) in women and of 9.30 points (95%CI:10.2,8.36) in men.

**Table 3:** Multivariable model: Differences between men and women in the sensitivity to determinants of physical functioning

	**Men**			**Women**			**Interaction**	***P*-value**
**Multivariable model**	Beta	95%CI		Beta	95%CI		Factor*sex	
Age	-0.18	(-0.24	-0.13)	-0.24	(-0.28	-0.19)	-0.05	0.18
**Socio-demographic determinants**								
Education								
Middle vs high	**-1.56**	(-3.03	-0.10)	0.50	(-1.15	2.14)	**2.34**	**0.04**
Low vs high	**-4.97**	(-6.44	-3.50)	**-1.99**	(-3.50	-0.49)	**3.33**	**0.00**
Work status (no vs yes)	**-4.13**	(-5.32	-2.95)	**-3.46**	(-4.47	-2.45)	-0.54	0.74
Living situation (alone vs together)	0.27	(-1.37	1.90)	**-1.82**	(-3.20	-0.50)	-1.29	0.19
**Lifestyle-related determinants**								
Alcohol consumption	0.52	(-0.45	1.48)	**1.45**	(0.33	2.58)	-0.33	0.74
(> 1 glass/day vs ≤ 1 glass/day)								
Current smoking status (yes vs no)	**-1.84**	(-3.06	-0.62)	-0.55	(-1.74	0.64)	1.79	**0.04**
Physical activity (h/week)	**0.10**	(0.07	0.12)	**0.06**	0.03	0.08	-0.02	0.23
Sleep hours								
Short vs normal	**-1.15**	(-2.24	-0.06)	-0.61	(-1.72	0.50)	0.78	0.34
Long vs normal	**-3.43**	(-5.20	-1.67)	**-4.72**	(-6.39	-3.04)	-1.58	0.21
BMI (kg/m2)	**-0.80**	(-0.96	-0.65)	**-0.85**	(-0.97	-0.74)	-0.06	0.78
**Health-related determinants**								
Chronic diseases								
One vs none	**-5.49**	(-6.43	-4.54)	**-6.22**	(-7.14	-5.32)	-0.69	0.35
More than one vs none	**-12.5**	(-14.2	-10.9)	**-11.4**	(-13.2	-9.65)	0.85	0.51
Pain								
Mild vs none	**-9.30**	(-10.2	-8.36)	**-10.9**	(-11.8	-9.87)	**-1.45**	**0.05**
Medium to severe vs none	**-23.2**	(-24.6	-21.9)	**-24.9**	(-26.1	-23.6)	-0.70	0.38
Poor mental health (MHI-5)	**-4.90**	(-6.25	-3.55)	**-3.21**	(-4.33	-2.08)	**1.81**	**0.02**

*Abbreviations: 95%CI* = *95% Confidence Interval, BMI* = *Body Mass Index*

*Note:* Bold is significant (for beta variable p < 0.05, for beta interaction p < 0.10)

*Note:* All interactions of sex*determinant (indicated by row) are individually tested in the full model including all variables

The differences between men and women in sensitivity were in general similar for adults above and below 55 years of age (data not shown). Exceptions were: a higher sensitivity to pain among women and to mental health problems among men were only significant among older adults, the sex-specific role of education was more pronounced among those < 55 years at baseline and the sex-specific role of work was only significant among adults aged < 55 years.

### Differences between men and women in exposure

The largest effect of a difference between men and women in exposure was found for pain: a larger sex difference was observed in the multivariable model including the determinant pain (-5.41, 95%CI: -6.41,-4.42) compared to the model without pain (-3.28, 95%CI:-4.13,-2.43) Table 4. So, adjusting for pain decreased the sex difference by (2.13/3.28) 65.0%, therefore pain contributes to the more reported limitations in physical functioning among women. In addition, women more often had a low educational level, no paid job and were alcohol abstainers, each decreasing around 10% of the beta for the sex difference in physical functioning and therefore contributing. In contrast, men were on average more exposed to a higher BMI and lower levels of physical activity compared to women, contributing to more reported limitations in physical functioning among men and therefore suppressing the sex difference.

**Table 4:** Multivariable model: Difference between men and women in exposure to determinants of physical functioning

Variable	Beta sex (women)^a^	95% CI		% Change Beta sex^b^
Full model (includes all variables)	-3.28	(-4.13	-2.43)	Reference
**Socio-demographic determinants**				
- Education (low/medium vs high)	-3.67	(-4.52	-2.82)	**- 11.9%**
- Work status (no vs yes)	-3.72	(-4.57	-2.87)	**- 13.5%**
- Living situation (alone vs together)	-3.30	(-4.15	-2.46)	- 0.6%
**Lifestyle-related determinants**				
- Alcohol consumption (> 1 glass/day vs ≤ 1 glass/day)	-3.45	(-4.28	-2.62)	**- 5.2%**
- Current smoking status (yes vs no)	-3.28	(-4.14	-2.43)	- 0.1%
- Physical activity (h/week)	-2.97	(-3.80	-2.14)	** + 9.5%**
- Sleep hours (long/short vs normal)	-3.26	(-4.11	-2.40)	+ 0.8%
- BMI (kg/m2)	-2.81	(-3.68	-1.94)	** + 14.2%**
**Health-related determinants**				
- Chronic diseases (one/two/more vs none)	-3.26	(-4.14	-2.39)	+ 0.5%
- Pain (mild/medium to severe vs none)	-5.41	(-6.41	-4.42)	**- 65.0% **^**c**^
- Mental health (poor vs good)	-3.41	(-4.26	-2.55)	- 3.9%

*Abbreviations: 95%CI* = *95% Confidence Interval, BMI* = *Body Mass Index*

*Note:* Each row represents the full model including all variables, age, age2 and sex excluding the indicated variable (-)

*Note:* Bold is significant (percentage change > 5%)

^a^Represents the sex difference (women versus men) in physical functioning

^b^Percentage change = 1—(Beta “sex” full model minus indicated variable / Beta “sex” full model), interpretation: A negative percentage change means this factor (partly) explains the lower physical functioning score among women compared to men (i.e. the sex difference decreases when adjusted for this determinant) and a positive percentage change suggest that this factor supresses the lower physical functioning score among women compared to men because after the adjustment the sex difference increases

^c^To illustrate: A larger sex difference (-5.41) was observed in the multivariable model including the risk factor pain compared to the model without pain (-3.28). So, adjusting for pain decreased the lower physical functioning score among women compared to men by (2.13/3.28) 65.0%

The differences between men and women in exposure were in general similar between adults above and below 55 years of age (data not shown). There were some differences in the relative size of contribution: work status contributed more among adults below versus above 55 years of age, whereas BMI suppressed more among those below 55 years. In exception, alcohol consumption only contributed to the sex difference among adults below 55 years.

#### Discussion

To summarize, our findings confirm that women report a poorer physical functioning compared to men (6.55 lower on average). This is similar to an older Finish population (mean age 61 years), where a mean difference of 6.67 points in the physical functioning score between men and women was found [23]. Other studies that use different measures to evaluate self-reported physical functioning (mainly ADL-focused measures) also show that women systematic report more limitations in physical functioning compared to men [1–4]. In addition, our results show that men and women have similar determinants of physical functioning. In exception, older women have a higher sensitivity to pain while men have a higher sensitivity to current smoking, a low educational level and mental health problems in association with physical functioning. Men and women more often differed in their exposure to determinants of poor physical functioning, where men were on average more exposed to a higher BMI and lower levels of physical activity, while women were higher exposed to pain, low alcohol consumption, lower educational level and no paid work, contributing to the more reported limitations in physical functioning among women.

Pain is the most important contributing determinant due to both a higher sensitivity and a higher exposure among (older) women compared to men. The association between pain and physical functioning has been found previously and determinants for pain seem to be quite similar to determinants associated with physical functioning [24]. In line with the current findings, pain is well-known to be more prevalent and more severe among women compared to men [13]. The type or origin of pain has not been measured in our study and can therefore not be disentangled, however, the contributing role of a higher exposure to pain does not differ between adult women or older women, suggesting a limited role of menstrual pain. The mechanisms underlying the association between pain and physical functioning are not fully understood [25]. Future studies on the link between pain and physical functioning should take differences between men and women into account to better understand the role of sex and/or gender in this association.

The current results suggest that tackling the health-related determinant pain may result in the reduction of the difference between older men and women in physical functioning. One study on the effect of a multimodal pain management program on pain-related physical disabilities in daily life suggested that older women (aged 40–60 years) improved more compared to men [26]. Preventing pain is, however, not an easy task, nor is pain management. Previous studies indicate that there are sex and gender differences in essential elements of pain treatment, suggesting the need for a specific approach for men and women. For example women use social support to cope with pain, while men seek behavior distraction [27]. To date, however, a men and women specific approach for pain treatment and management is often lacking. Besides pain also ‘poor mental health’ is a health issue associated with physical functioning, but in contrast to pain the association is stronger among men compared to women. Because men seem to be more sensitive to poor mental health, this does not contribute to the difference in physical functioning with more limitations among women, but it does emphasize the relevance of a specific approach for men and women. No earlier studies on the specific association for men and women between mental health and physical functioning were found.

This exploration on differences between men and women in sensitivity or exposure to determinants of self-reported physical functioning provides almost no clues for reducing the sex difference in physical functioning by a prevention strategy directed to modifiable lifestyle-related determinants. Though physical functioning in general may benefit from a healthier lifestyle, this might not reduce the more reported limitations in physical functioning among women compared to men. The prevalence of lifestyle-related determinants, in particular a higher BMI, is higher among middle aged men. A higher BMI is associated with a greater physical decline [2], but most of these studies on the relationship between BMI and physical functioning do not present data specific for men and women. A difference between men and women in the role of smoking for physical functioning was reported in the current study, in line with a recent review which suggests that the relative risk of smokers for impaired function is higher among men than among women [28]. For the association between physical activity and health-related quality of life, Liao et al. (2020) found a stronger positive association among men [29]. The current findings did not reach a significant difference between men and women in the association of physical activity with physical functioning, although a trend was observed.

Of the socio-demographic determinants, ‘living alone’ did not contribute to the difference between men and women in physical functioning. In contrast, both the quantity and quality of social support have been shown to have a greater impact on the well-being of women compared to men [30]. It is therefore recommended to include more extensive indicators for social contacts and social support in future studies, which were not included at baseline in the Doetinchem Cohort Study. The findings that educational level and work status were associated with physical functioning are in line with previous studies [31], but its contributing role for predominance of women in physical functioning limitations is not reported before. Intervention strategies aiming for equal opportunities regarding the work field, for example regarding promotion strategies, might decrease the difference in physical functioning between men and women.

The strengths of the current study include the use of a broad range of determinants, a large-scale data set and the use of a commonly used measure of physical functioning. The physical functioning domain of the SF-36 focuses on the ability to carry out specific physical activities, and to a lesser extent on daily routine and instrumental activities, and is therefore an adequate tool to measure physical functioning in an ageing cohort [23]. While interpreting the findings of the current study, some limitations should be taken into account. First, there is some selection bias towards a healthier and younger population, commonly found in cohort studies [14]. However, the associations between the determinants of chronic health problems are usually not likely to be affected by attrition [32]. In addition, there was no significant difference found in the response bias between men and women, and thus not affecting the current study of differences between men and women [32]. However, since women have a higher life expectancy this could lead to an overrepresentation of older women with poor physical functioning compared to men. Second, both the physical functioning and pain measures are self-reported and therefore bias may be gender specific: women may report more disability compared to men as a result of their increased subjective impression of functional loss and men are socially conditioned to neglect pain and disease [33]. However, it has also been demonstrated that women have similar degrees of self-reported limitation in physical functioning as men of the same age, health, and physical abilities [34]. In addition, the lower score among women compared to men is both found using self-report and performance-based measures [35]. Third, some relevant determinants for physical functioning were not included in the current study, e.g. cognitive functioning, vision and social contacts [5]. Cognitive functioning was not incorporated in our analyses, since cognition was measured in less than half of the sample and vision and social contacts were not included in the baseline measurement. It is recommended for future studies to take these variables into account. Fourth, a statistical approach was used to investigate ‘sensitivity’ and ‘exposure’ to determinants, but it is probably impossible to completely disentangle those two. Paradoxically, more limitations in physical functioning among women compared to men goes together with a lower mortality among women [4]. Other explanations for this sex/gender-health paradox than the determinant approach explored here, include sex differences in physiological aging such as differences between in chronic inflammation, in immunosenescence and in genes [11].

#### Conclusions

For the aim of explaining and reducing the ‘female disadvantage’ in physical functioning, the ‘sensitivity and exposure’ approach shows that in particular the difference between man and women in pain seems relevant. Hence, although an effective prevention program focusing on lifestyle-related determinants is known to improve physical functioning, it might not reduce the difference between men and women in physical functioning, where women report more limitations. Differences between men and women in physical functioning and health in general are persistent and require more attention from research, prevention and disease management in order to contribute to more equal opportunities in health.

## Supplementary Information

**Additional file 1: Figure S1.** Flowchart of the Doetinchem cohort Study, analyses of physical functioning (subscale SF-36) rounds and the final study population.

## Data Availability

Data cannot be shared publicly because of confidentiality. Data are available from the Doetinchem Cohort Study (contact via https://www.rivm.nl/doetinchem-cohort-studie/onderzoekers/aanvraag-gegevens-dcs and doetinchemstudie@rivm.nl) for researchers who meet the criteria for access to confidential data. The data underlying the results presented in the study are available from the Doetinchem Cohort Study. The Scientific Advisory Group ensures that proposals for the use of the Doetinchem Cohort Study data do not violate privacy regulations and are in keeping with informed consent that is provided by all participants. The authors of this study do not have any special access privileges to the data underlying this study that other researchers would not have.

## Abbreviations

BMI: Body Mass Index
WHO: World Health Organization
