# Supplementary figures and images for: Exploring the difference between men and women in physical functioning: How do sociodemographic, lifestyle- and health-related determinants contribute?
Source: BMC Geriatr. 2022 Jul 22;22:610. doi: 10.1186/s12877-022-03216-y (PMC9306105; doi:10.1186/s12877-022-03216-y)

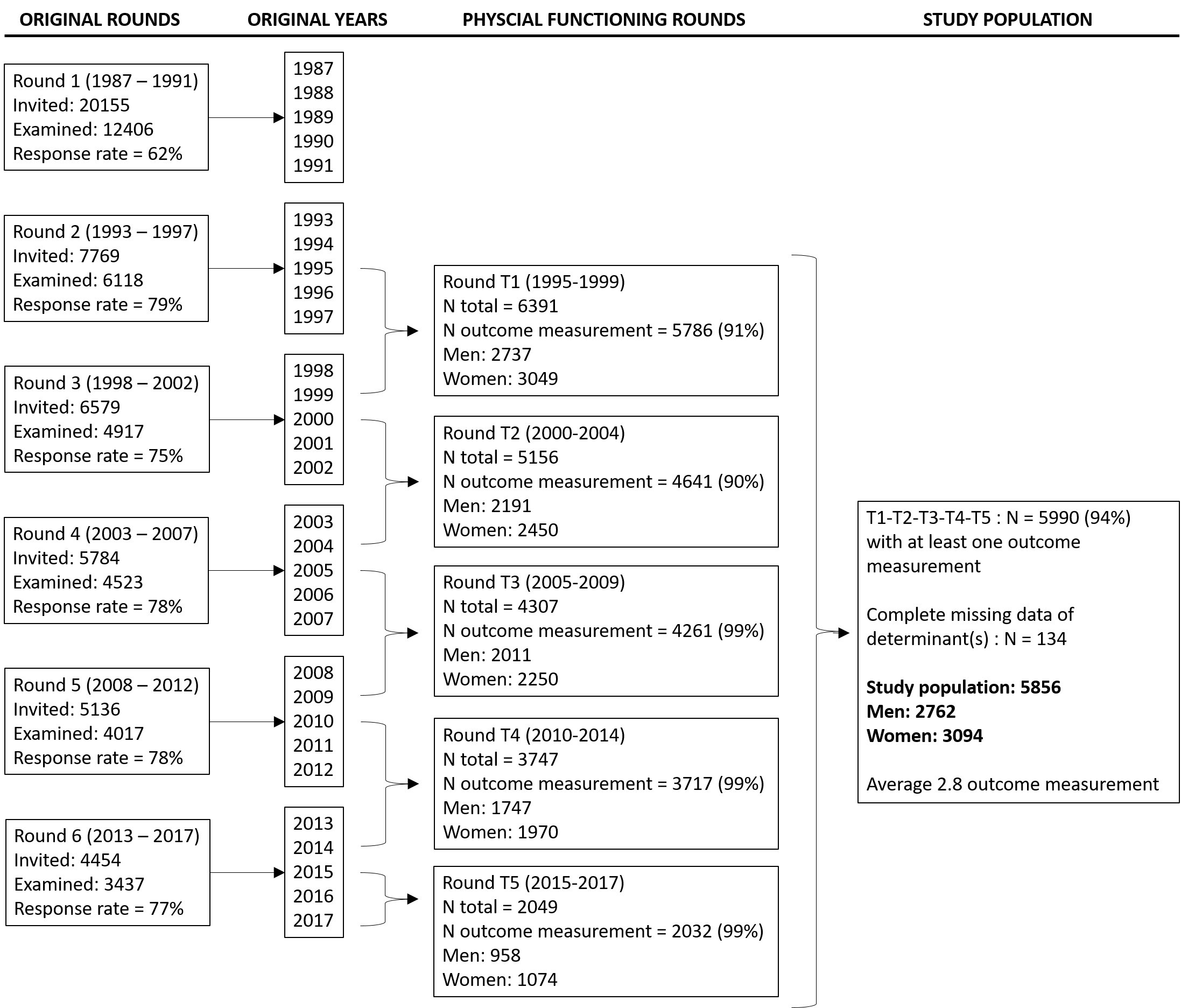

Supplement: Supplementary file 1 — Additional file 1: Figure S1. Flowchart of the Doetinchem cohort Study, analyses of physical functioning (subscale SF-36) rounds and the final study population. [file 12877_2022_3216_MOESM1_ESM.png]
